# Supplementary material for: Tailoring the evolution of BL21(DE3) uncovers a key role for RNA stability in gene expression toxicity
Source: Commun Biol. 2021 Aug 12;4:963. doi: 10.1038/s42003-021-02493-4 (PMC8361080; doi:10.1038/s42003-021-02493-4)
Supplement: Supplementary file 2 — Supplementary Information [file 42003_2021_2493_MOESM2_ESM.pdf]

Supplementary Information

Tailoring the evolution of BL21(DE3) uncovers a key role for RNA stability in gene expression toxicity

Sophia A. H. Heyde[1] and Morten H. H. Nørholm[1,2]

[1]Novo Nordisk Foundation Center for Biosustainability, Technical University of Denmark, Kemitorvet B220, DK-2800 Kgs. Lyngby, Denmark

[2]Correspondance to morno@biosustain.dtu.dk

Supplementary Figure 1:

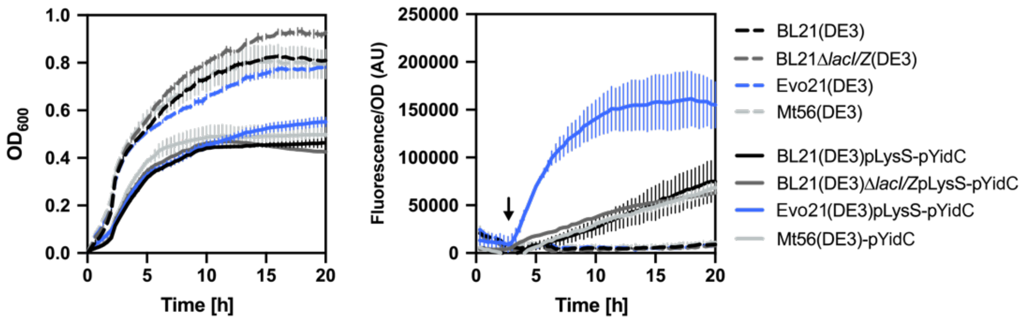

Growth phenotype and GFP fluorescence profile of BL21(DE3) and derivatives BL21(DE3)*)ΔlacI/Z*, Evo21(DE3) and Mt56(DE3) when grown for 20 h harbouring no expression vector compared to when carrying plasmids pLysS and pYidC. Induction of YidC-GFP production at OD600 = 0.3 via addition of IPTG is indicated (arrow).

Supplementary Figure 2:

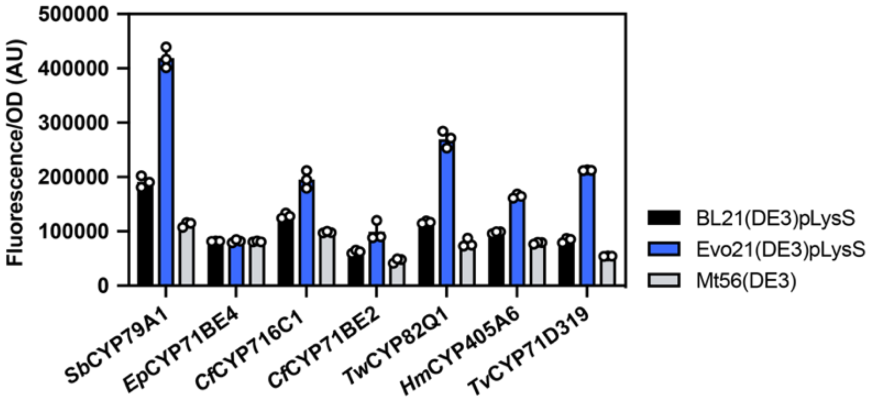

Expression of a small library of seven plant-derived P450 enzymes in Evo21(DE3) compared to BL21(DE3) and Mt56(DE3). Proteins are expressed from a pET/T7-derived vector, and production was induced via the addition of IPTG.

Supplementary Figure 3:

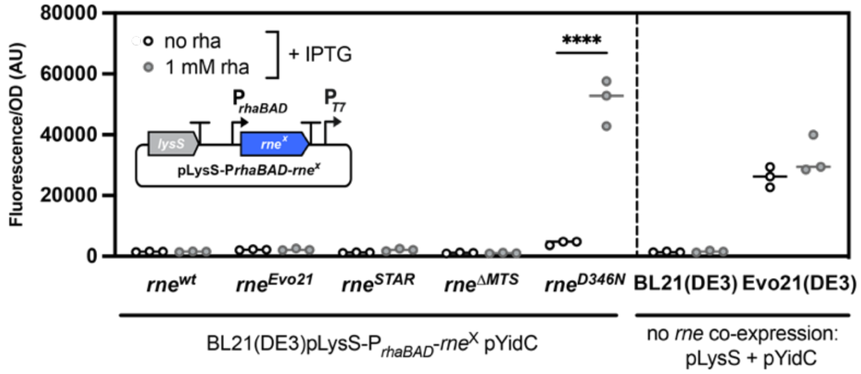

Co-expression of *rne* variants on auxiliary plasmid pLysS and *yidC-gfp* expression vector pYidC in BL21(DE3). RNase E production is under the control of a rhamnose promoter. YidC-GFP expression is induced via IPTG. MTS: membrane targeting sequence.

Supplementary Figure 4:

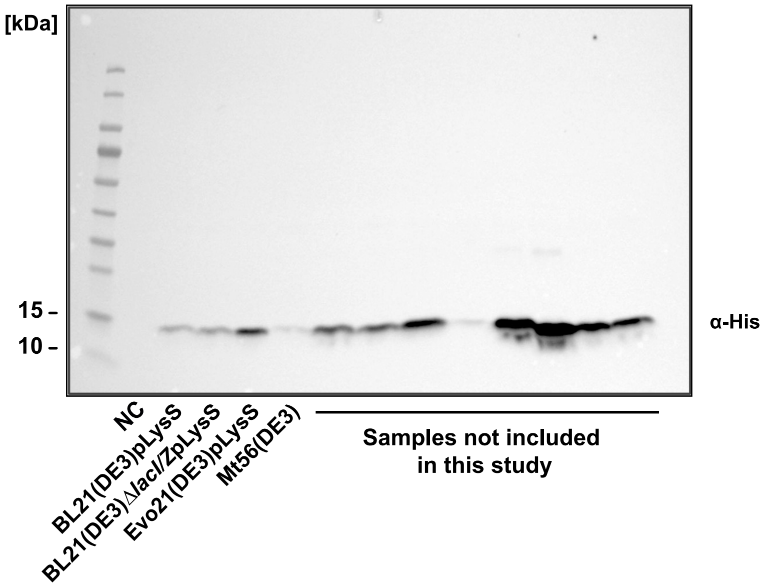

Uncut western blot corresponding to Figure 2d in the main manuscript text. Western blot showing the expression of a camelid-derived single-chain antibody (nanobody) in Evo21(DE3) and control strains. Samples were normalised to cell density before loading.

Supplementary Table 1: Proteins used in this study

**Non-toxic**				
**Protein**	**Organism**	**Expression vector**	**Corresponding publication**	**Additional information**
frGFP	*Escherichia coli, Aequorea victoria*	pET/T7	Bradley et al., 2000	Folding reporter GFP; *malE-gfp* fusion
nanobody	*Cameloids*	pET/T7	Rennig et al., 2017	Single-chain cameloid antibody fragment
**Toxic**				
**Protein**	**Organism**	**Expression vector**	**Corresponding publication**	**Additional information**
YijD	*Escherichia coli*	pET/T7	Daley et al., 2005	Length: 120, Function: Unknown, TMHMM(#TM): 4, TMHMM(S3score): 0,0381944444444444
LspA	*Escherichia coli*	pET/T7	Daley et al., 2005	Length: 165, Function: Biogenesis, TMHMM(#TM): 4, TMHMM(S3score): 0,0666666666666667
YgiH	*Escherichia coli*	pET/T7	Daley et al., 2005	Length: 206, Function: Unknown, TMHMM(#TM): 5, TMHMM(S3score): 0,0208333333333333
CysZ	*Escherichia coli*	pET/T7	Daley et al., 2005	Length: 254, Function: Transport/influx, TMHMM(#TM): 4, TMHMM(S3score): 0,0625
HtpX	*Escherichia coli*	pET/T7	Daley et al., 2005	Length: 294, Function: Unknown, TMHMM(#TM): 4, TMHMM(S3score): 0,0604166666666667
YihG	*Escherichia coli*	pET/T7	Daley et al., 2005	Length: 311, Function: Unknown, TMHMM(#TM): 2, TMHMM(S3score): 0,03125
WzzE	*Escherichia coli*	pET/T7	Daley et al., 2005	Length: 349, Function: Lipid, TMHMM(#TM): 2, TMHMM(S3score): 0,06875
YdhP	*Escherichia coli*	pET/T7	Daley et al., 2005	Length: 390, Function: Transport/efflux, TMHMM(#TM): 12, TMHMM(S3score): 0,0208333333333333
HybB	*Escherichia coli*	pET/T7	Daley et al., 2005	Length: 393, Function: Metabolism, TMHMM(#TM): 10, TMHMM(S3score): 0,05
PqiA	*Escherichia coli*	pET/T7	Daley et al., 2005	Length: 418, Function: Unknown, TMHMM(#TM): 8, TMHMM(S3score): 0,0319444444444444
Kch	*Escherichia coli*	pET/T7	Daley et al., 2005	Length: 418, Function: Channel, TMHMM(#TM): 6, TMHMM(S3score): 0,0277777777777778
UraA	*Escherichia coli*	pET/T7	Daley et al., 2005	Length: 430, Function: Transport/influx, TMHMM(#TM): 12, TMHMM(S3score): 0,00486111111111111
BrnQ	*Escherichia coli*	pET/T7	Daley et al., 2005	Length: 440, Function: Transport/influx, TMHMM(#TM): 12, TMHMM(S3score): 0,0159722222222222

PheP	*Escherichia coli*	pET/T7	Daley et al., 2005	Length: 459, Function: Transport/influx, TMHMM(#TM): 12, TMHMM(S3score): 0,0208333333333333
NarK	*Escherichia coli*	pET/T7	Daley et al., 2005	Length: 464, Function: Transport/efflux, TMHMM(#TM): 12, TMHMM(S3score): 0,0381944444444444
HydH	*Escherichia coli*	pET/T7	Daley et al., 2005	Length: 466, Function: Signaling, TMHMM(#TM): 2, TMHMM(S3score): 0,0423611111111111
WzxC	*Escherichia coli*	pET/T7	Daley et al., 2005	Length: 493, Function: Lipid, TMHMM(#TM): 12, TMHMM(S3score): 0,00972222222222222
CydA	*Escherichia coli*	pET/T7	Daley et al., 2005	Length: 524, Function: Metabolism, TMHMM(#TM): 9, TMHMM(S3score): 0,0465277777777778
YqiK	*Escherichia coli*	pET/T7	Daley et al., 2005	Length: 554, Function: Unknown, TMHMM(#TM): 1, TMHMM(S3score): 0,0673611111111111
FdoI	*Escherichia coli*	pET/T7	Daley et al., 2005	Length: 212, Function: Metabolism, TMHMM(#TM): 4, TMHMM(S3score): 0,0673611111111111
ProW	*Escherichia coli*	pET/T7	Daley et al., 2005	Length: 355, Function: Transport/influx, TMHMM(#TM): 6, TMHMM(S3score): 0,0222222222222222
ShiA	*Escherichia coli*	pET/T7	Daley et al., 2005	Length: 439, Function: Transport/influx, TMHMM(#TM): 12, TMHMM(S3score): 0,0291666666666667
YliF	*Escherichia coli*	pET/T7	Daley et al., 2005	Length: 443, Function: Unknown, TMHMM(#TM): 2, TMHMM(S3score): 0,0590277777777778
FucP	*Escherichia coli*	pET/T7	Daley et al., 2005	Length: 439, Function: Transport/influx, TMHMM(#TM): 12, TMHMM(S3score): 0,00763888888888889
*Sb*CYP79A1	*Sorghum bicolor*	pET/T7	Vazquez-Albacete et al., 2016	Membrane Bound Plant Cytochrome P450
*Ep*CYP71BE4	*Euphorbia peplus*	pET/T7	Vazquez-Albacete et al., 2016	Membrane Bound Plant Cytochrome P450
*Cf*CYP716C1	*Coleus forskohlii*	pET/T7	Vazquez-Albacete et al., 2016	Membrane Bound Plant Cytochrome P450
*Cf*CYP71BE2	*Coleus forskohlii*	pET/T7	Vazquez-Albacete et al., 2016	Membrane Bound Plant Cytochrome P450
*Tw*CYP82Q1	*Tripterygium willfordii*	pET/T7	Vazquez-Albacete et al., 2016	Membrane Bound Plant Cytochrome P450
Hm CYP405A6	*Heliconius Melpomene*	pET/T7	Vazquez-Albacete et al., 2016	Membrane Bound Plant Cytochrome P450
Tv CYP71D319	*Thapisa villosa*	pET/T7	Vazquez-Albacete et al., 2016	Membrane Bound Plant Cytochrome P450

Supplementary Table 2: MAGE oligos used in this study

Oligo	Genomic location	Info	Sequence
**1**	*argE*	*tetA* integration (fw primer)	gtttggcaacggtgccggatgcggcgcgagcgccttatccg gcctacgttagaaatcatccttagcgaaa
**2**	*argE*	*tetA* integration (rv primer)	ataaatactgcatgaatattgatactatcatgaccagaggtgtg tcaacattgcttattaatcatccggctc
**3**	*argE*	*tetA* removal (MAGE oligo)	attgaatttgttgcgagttcctggaacaggctgcacttccacatt gaagctcaaatctttaaaccagtccgccagcagagtaattaaa tctgcattgctt
**4**	*fecB*	*tetA* integration (fw primer)	gctgcaacgggaagcccccacagcagcaccgggtgttttat cgcggtcatagaaatcatccttagcgaaagctaagga
**5**	*fecB*	*tetA* integration (rv primer)	ttctgatgtgttaacgcccggcttgccgggcttttagctggaat gtgattttgcttattaatcatccggctcgt
**6**	*fecB*	*tetA* removal (MAGE oligo)	ctctctttatgttgttccaggcgtgcctgcatctctcgctttttacc caccatttcgccgatgatagccgcaggttgcaaattttcagcg taggtttcgt
**7**	*rne*	*tetA* integration (fw primer)	accggacgtacacgttcttcctgttcggtttcctgaacagattg ctcttcttgcttattaatcatccggctcgtataatgt
**8**	*rne*	*tetA* integration (rv primer)	gtaatgatgataaacgtcaggcgcaacaagaagcgaaggc gctgaatgtttagagaaatcatccttagcgaaagctaagg
**9**	*rne*	*tetA* removal (MAGE oligo)	gtaatgatgataaacgtcaggcgcaacaagaagcgaaggc gctgaatgttgaagagcaaggtaatgactccaacttattgata gtgttttatgttcagataatgcccgatgactttgtcatgcagctc caccgattttgatctgttcaggaaaccgaacaggaagaacgt gtacgtccggttcagccgcgtcgtaaacag
