# Supplementary material for: Tailoring the evolution of BL21(DE3) uncovers a key role for RNA stability in gene expression toxicity
Source: Commun Biol. 2021 Aug 12;4:963. doi: 10.1038/s42003-021-02493-4 (PMC8361080; doi:10.1038/s42003-021-02493-4)
Supplement: Supplementary file 5 — Reporting Summary [file 42003_2021_2493_MOESM5_ESM.pdf]

nature research

Corresponding author(s): Morten Nørholm

Last updated by author(s): 28-06-2021

Reporting Summary

Nature Research wishes to improve the reproducibility of the work that we publish. This form provides structure for consistency and transparency in reporting. For further information on Nature Research policies, see our Editorial Policies and the Editorial Policy Checklist.

Statistics

For all statistical analyses, confirm that the following items are present in the figure legend, table legend, main text, or Methods section.

n/a	Confirmed	
[x]	[ ]	The exact sample size (*n*) for each experimental group/condition, given as a discrete number and unit of measurement
[ ]	[x]	A statement on whether measurements were taken from distinct samples or whether the same sample was measured repeatedly
[ ]	[x]	The statistical test(s) used AND whether they are one- or two-sided<br>*Only common tests should be described solely by name; describe more complex techniques in the Methods section.*
[ ]	[x]	A description of all covariates tested
[ ]	[x]	A description of any assumptions or corrections, such as tests of normality and adjustment for multiple comparisons
[ ]	[x]	A full description of the statistical parameters including central tendency (e.g. means) or other basic estimates (e.g. regression coefficient) AND variation (e.g. standard deviation) or associated estimates of uncertainty (e.g. confidence intervals)
[x]	[ ]	For null hypothesis testing, the test statistic (e.g. *F*, *t*, *r*) with confidence intervals, effect sizes, degrees of freedom and *P* value noted<br>*Give P values as exact values whenever suitable.*
[x]	[ ]	For Bayesian analysis, information on the choice of priors and Markov chain Monte Carlo settings
[x]	[ ]	For hierarchical and complex designs, identification of the appropriate level for tests and full reporting of outcomes
[x]	[ ]	Estimates of effect sizes (e.g. Cohen's *d*, Pearson's *r*), indicating how they were calculated

Our web collection on statistics for biologists contains articles on many of the points above.

Software and code

Policy information about availability of computer code

Data collection	none
Data analysis	none

For manuscripts utilizing custom algorithms or software that are central to the research but not yet described in published literature, software must be made available to editors and reviewers. We strongly encourage code deposition in a community repository (e.g. GitHub). See the Nature Research guidelines for submitting code & software for further information.

Data

Policy information about availability of data

All manuscripts must include a data availability statement. This statement should provide the following information, where applicable:

- Accession codes, unique identifiers, or web links for publicly available datasets
- A list of figures that have associated raw data
- A description of any restrictions on data availability

All whole genome sequencing data connected to this study has been deposited to the Sequence Read Archive (SRA) and can be accessed via BioProject ID PRJNA741995. All other data will be made available upon reasonable request to the corresponding author. The uncropped and unedited western blot image corresponding to Figure 2d is included as Supplementary Figure 4.

Field-specific reporting

Please select the one below that is the best fit for your research. If you are not sure, read the appropriate sections before making your selection.

☒ Life sciences ☐ Behavioural & social sciences ☐ Ecological, evolutionary & environmental sciences

For a reference copy of the document with all sections, see nature.com/documents/nr-reporting-summary-flat.pdf

Life sciences study design

All studies must disclose on these points even when the disclosure is negative.

Sample size	All experiment have been performed in biological triplicates.
Data exclusions	No data was excluded from the analysis.
Replication	All attempts of replication were successful.
Randomization	This is not relevant to this study.
Blinding	This is not relevant to this study.

Reporting for specific materials, systems and methods

We require information from authors about some types of materials, experimental systems and methods used in many studies. Here, indicate whether each material, system or method listed is relevant to your study. If you are not sure if a list item applies to your research, read the appropriate section before selecting a response.

Materials & experimental systems

n/a	Involved in the study	
☐	☒	Antibodies
☒	☐	Eukaryotic cell lines
☒	☐	Palaeontology and archaeology
☒	☐	Animals and other organisms
☒	☐	Human research participants
☒	☐	Clinical data
☒	☐	Dual use research of concern

Methods

n/a	Involved in the study	
☒	☐	ChIP-seq
☒	☐	Flow cytometry
☒	☐	MRI-based neuroimaging

Antibodies

Antibodies used	Antibodies have been used for western blotting: primary antibody (anti-8xHis mouse antibody (05-949, LOT: 3033896, 1:1000, Merck Millipore, Darmstadt, Germany) and secondary antibody (HRP-conjugated rabbit anti-mouse IgG (AP160P, 1:10000, Merck Millipore, Darmstadt, Germany)).
Validation	Primary antibody: Anti-Histidine Tagged Antibody, clone HIS.H8 (Purified mouse monoclonal IgG2b in buffer containing PBS, pH 7.4 with 0.05% sodium azide. Quality Level MG100)<br>His-Tag is a series of at least 6 histidine residues used as a tag motif at the N- or C-terminus of recombinant proteins. The relatively small size of this tag vs. other commonly used tags such as GST (30 kDa) makes it less likely to interfere with target protein structure or function. Its small size also makes it more suitable for use under denaturing conditions. His tags are most often used for affinity purification and binding assays. His tags bind to nickel or cobalt ions with high affinity, and this interaction with metal ions is the basis of many his-tag based affinity media. The His Tag epitope is widely used to detect expression of recombinant proteins in bacterial, yeast, insect,and mammalian systems.
